# Manuka Honey/2-Hydroxyethyl Methacrylate/Gelatin Hybrid Hydrogel Scaffolds for Potential Tissue Regeneration

**DOI:** 10.3390/polym15030589

**Published:** 2023-01-24

**Authors:** Simonida Lj. Tomić, Jovana S. Vuković, Marija M. Babić Radić, Vuk. V. Filipović, Dubravka P. Živanović, Miloš M. Nikolić, Jasmina Nikodinovic-Runic

**Affiliations:** 1 University of Belgrade, Faculty of Technology and Metallurgy, Karnegijeva 4, 11000 Belgrade, Serbia; 2 University of Belgrade, Institute of Molecular Genetics and Genetic Engineering, Vojvode Stepe 444a, 11000 Belgrade, Serbia; 3 University of Belgrade, Faculty of Medicine, Department of Dermatology and Venereology, Pasterova 2, 11000 Belgrade, Serbia; 4 University of Belgrade, University Clinical Center of Serbia, Clinic of Dermatology and Venereology, Pasterova 2, 11000 Belgrade, Serbia

**Keywords:** Manuka honey, 2-hydroxyethyl methacrylate, gelatin, pH- and temperature-dependent swelling, in vitro degradation, in vitro biocompatibility

## Abstract

Scaffolding biomaterials are gaining great importance due to their beneficial properties for medical purposes. Targeted biomaterial engineering strategies through the synergy of different material types can be applied to design hybrid scaffolding biomaterials with advantageous properties for biomedical applications. In our research, a novel combination of the bioactive agent Manuka honey (MHo) with 2-hydroxyethyl methacrylate/gelatin (HG) hydrogel scaffolds was created as an efficient bioactive platform for biomedical applications. The effects of Manuka honey content on structural characteristics, porosity, swelling performance, in vitro degradation, and in vitro biocompatibility (fibroblast and keratinocyte cell lines) of hybrid hydrogel scaffolds were studied using Fourier transform infrared spectroscopy, the gravimetric method, and in vitro MTT biocompatibility assays. The engineered hybrid hydrogel scaffolds show advantageous properties, including porosity in the range of 71.25% to 90.09%, specific pH- and temperature-dependent swelling performance, and convenient absorption capacity. In vitro degradation studies showed scaffold degradability ranging from 6.27% to 27.18% for four weeks. In vitro biocompatibility assays on healthy human fibroblast (MRC5 cells) and keratinocyte (HaCaT cells) cell lines by MTT tests showed that cell viability depends on the Manuka honey content loaded in the HG hydrogel scaffolds. A sample containing the highest Manuka honey content (30%) exhibited the best biocompatible properties. The obtained results reveal that the synergy of the bioactive agent, Manuka honey, with 2-hydroxyethyl methacrylate/gelatin as hybrid hydrogel scaffolds has potential for biomedical purposes. By tuning the Manuka honey content in HG hydrogel scaffolds advantageous properties of hybrid scaffolds can be achieved for biomedical applications.

## 1. Introduction

The use of agents derived from nature that possess medicinal properties has always been an intriguing topic. Honey is a bioactive agent, and its healing properties have been recognized for centuries [1,2,3]. Manuka honey (MHo) stands out due to the specific Manuka factor (UMF), which provides antioxidant, antibacterial, and anti-inflammatory effects [4,5,6,7]. MHo is isolated from *Leptospermum scoparium* nectar species that grow in New Zealand with a pH range of 3.5–4.5. Honey features may influence increased macrophage stimulation, facilitating tissue regeneration and minimizing bacterial infections [4,8,9,10,11,12,13,14]. Manuka honey has proven to be a very effective agent in the treatment of wounds [1,2].

Regenerative medicine is a field that functions with specific treatment methods to improve healing outcomes and has been fueled by advances in biomedical engineering in recent decades. Strategies and platforms for healing including biofunctional scaffolds are unique in their purposes as “powerful tools” for restoring, maintaining, and revitalizing damaged tissues and organs. Such actions have a great impact on the entire medical field and healthcare systems [15]. Hydrogels are polymeric networks suitable for the biomedical engineering of scaffolding biomaterials. They possess distinct appropriate 3D structures and mechanical strength for the cells in the engineered tissues and may imitate the extracellular matrix [16,17,18,19,20,21]. Tunable hydrogel properties such as the amount of water/biological fluid represent an ideal environment to assist in cell regrowth. The combination of polymeric components of synthetic and natural origin is a possible biomaterial engineering strategy to achieve the convenient performance of hydrogels for biomedical tissue engineering applications [22,23,24,25]. Scaffolding biomaterials are suitable for regenerative medicine and biomedical engineering strategies and, as such, influence the restoration and improvement of tissue and organ function by providing an appropriate basis for cell growth, proliferation, and differentiation [26]. Scaffolds can be designed in many modes, such as hydrogel, fibrous, acellular, hybrid, and porous scaffolds [26,27,28,29,30,31,32]. Among these various types of scaffolds, hydrogel porous scaffolds possess advantages that can be used for biomedical tissue engineering due to specific features such as suitable mechanical strength and 3D porosity, which is a beneficial basis for cell penetration and population [27]. Scaffolds must exhibit appropriate biocompatible behavior for cell functions and functioning, as well as the re-establishment of tissue structural integrity and function [14,28,29,30,31,32]. They also should be a barrier to infections and be degradable [14,28,29,30,31,32].

One of the most versatile scaffolding materials and multifunctional polymers of natural origin is gelatin [33,34,35,36]. Its backbone consists of positively charged free carboxyl and amino groups. The known arginine–glycine–aspartic acid (RGD) sequence advances cell attachment and proliferation [33,34,35,36]. Gelatin is a very convenient component for scaffold preparations due to its availability, water solubility, and suitable immunogenic response. Scaffolds based on gelatin can be enzymatically/hydrolytically degraded in such a way as to create free space for the formation of a new extracellular matrix (ECM). Gelatin-based hydrogel scaffolds are adjustable for three-dimensional biomedical engineering applications [33,36,37,38,39,40]. A favorable component for designing various hydrogel types and hydrogel scaffolding biomaterials is 2-hydroxyethyl methacrylate (HEMA) as a versatile monomer. Polymeric biomaterials based on HEMA are successfully used in wound dressings and healing, controlled drug release systems, regenerative medicine, and other medical fields [41,42,43,44,45,46,47]. HEMA-based hydrogels possess suitable and easily tunable beneficial features such as morphology, hydrophilicity, absorption capacity, biocompatibility, and tissue-like mechanical behavior, enabling such biomedical applications [48,49,50].

In our study, we designed a hybrid hydrogel scaffolding platform consisting of 2-hydroxyethyl methacrylate/gelatin scaffolds loaded with Manuka honey. Honey content was varied to evaluate its effect on structural characteristics, porosity, pH- and temperature-dependent swelling performance, in vitro degradation, and in vitro biocompatibility in interaction with fibroblast and keratinocyte cell lines. Each component possesses specific properties and advantageous features, as shown by constructs. Manuka honey possesses plenty of beneficial biofunctionalities and bioactivities, which contribute to the overall quality of synthesized hybrid scaffolds.

## 2. Materials and Methods

### 2.1. Materials

2-Hydroxyethyl methacrylate (H), gelatin (G, Type A), ethylene glycol dimethacrylate (EGDMA), 1-ethyl-3-(3-dimethyl aminopropyl) carbodiimide hydrochloride (EDC), ammonium persulfate (APS), and N,N,N’,N’-tetramethylene diamine (TEMED) were purchased from Sigma-Aldrich, St. Louis, MO, USA. Manuka honey (MHo) was supplied by Haines Apiaries Ltd. (ArtisanHoney^®^, Kaitaia, New Zealand). RPMI-1640 medium and supplements for cell proliferation, as well as 3-(4,5-dimethylthiazol-2-yl)-2,5-diphenyltetrazolium bromide (MTT) reduction assay components, were purchased from Sigma-Aldrich, St. Louis, MO, USA. All syntheses were performed in deionized water. All experiments were performed using lab-produced, ultra-distilled water.

### 2.2. Hydrogel Scaffold Syntheses

**Figure 1:**
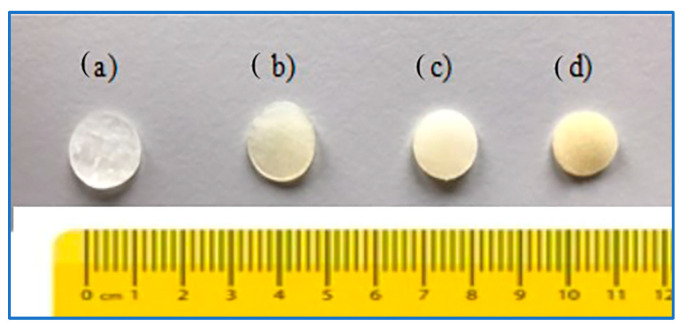
Photograph of fabricated hybrid hydrogel scaffolds (**a**) HG, (**b**) 10MHo/HG, (**c**) 20MHo/HG, and (**d**) 30MHo/HG.

Hybrid hydrogel scaffolds consisting of Manuka honey, 2-hydroxyethyl methacrylate, and gelatin were synthesized using polymerization/crosslinking reactions. An aqueous solution of gelatin was added to 2-hydroxyethyl methacrylate. The mixture was heated (40 °C) with intensive mixing to completely dissolve the gelatin. Honey was added to the homogeneous mixture, and mixing was continued until all the honey was dissolved. Manuka honey content was 10, 20, and 30 wt%. Then, a solution of agents for polymerization/crosslinking reactions (1% solution of APS, TEMED, and EDC) was added with intensive mixing until the mixture became a milky-white color and viscous. The reaction mixtures were transferred to a Petri dish, where the polymerization/crosslinking reactions took place at a temperature of −18 °C for 24 h. When the reactions were finished, the samples were cut into disc form and dried at room temperature to a constant weight. A photograph of the synthesized hybrid hydrogel scaffolds is shown in Figure 1 (the average dimensions of the samples are: diameter, 1 cm; thickness, 0.2 cm; weight, 0.2 g). The samples were placed in a deep freezer (−80 °C) and lyophilized (−55 °C) for further characterization.

The composition and marks of the hybrid hydrogel scaffold samples are presented in Table 1.

### 2.3. Hybrid Hydrogel Scaffold Characterization

#### 2.3.1. Fourier Transform Infrared Spectroscopy (FTIR)

Hydrogel scaffold structural characteristics were detected by the FTIR spectroscopy method recorded on a Thermo-Scientific Nicolet 6700 FTIR diamond crystal spectrometer using the attenuated total reflectance (ATR) sampling technique. FTIR spectra were recorded over the wavelength range of 700–4000 cm^−1^.

#### 2.3.2. Porosity Measurements

The porosity of hydrogel scaffolds was determined by the solvent replacement method. Glycerol (ρ = 1.2038 g/cm^3^) was used as a wetting medium. Dried hydrogels were submerged in glycerol for 24 h and weighed after removing excess glycerol from the surface:

$$\mathrm{Porosity}=\frac{\left(m_{\mathrm{glycerol}}-m_{i}\right)}{\rho V}*100$$

where m_i_ is the initial weight of the dry hydrogel, m_glycerol_ is the weight of the hydrogel with glycerol, ρ is the density of glycerol, and V is the volume of the hydrogel sample. All experiments were performed in triplicate.

#### 2.3.3. In Vitro pH- and Temperature-Dependent Swelling Studies

In vitro swelling studies were performed in a milieu with buffers mimicking biological fluids in a pH range of 2.20–8.0 at 37 °C and a temperature range of 25–39 °C in buffers of pH 7.40 and 5.50 to reveal swelling behavior important for biomedical applications. The amount of fluid absorbed as a function of time was measured gravimetrically. Swollen hydrogels were removed from the swelling medium at regular intervals and dried superficially with filter paper. They were weighed and placed in the same bath until a constant weight was reached for each sample in an equilibrium swelling state. The equilibrium degree of swelling (q_e_) is calculated using the following formula:

$$q_{e}=\frac{m_{e}-m_{0}}{m_{0}}$$

where m_e_ is the weight of a swollen hydrogel at equilibrium, and m_0_ is the weight of a dry gel [51,52]. All swelling experiments were performed in triplicate.

#### 2.3.4. In Vitro Degradation Study

The in vitro degradation process was tested by immersion of the dry gel samples in a buffer of pH 7.40 at 37 °C for four weeks. Samples were removed from the buffer at the estimated time, dried at 40 °C until a constant mass was reached, and weighed. Degradation is expressed by weight loss (%) calculated as the residual hydrogel weight percentage:

$$\mathrm{Weight}\;\mathrm{loss}\;\left(\%\right)=\frac{m_{t}}{m_{i}}*100$$

where m_i_ is the initial weight of the dry gel, and m_t_ is the weight of the dried gel sample at the measurement time. Scaffold degradation is expressed as a function of time. All degradation experiments were performed in triplicate.

### 2.4. In Vitro Biocompatibility Assay

The in vitro cytocompatibility of hybrid hydrogel scaffolds is evaluated to indicate potential biomedical applications [53]. Cytotoxicity/antiproliferative activity was measured for interaction scaffolding materials with human lung fibroblasts (MRC5 cell line) and keratinocytes (HaCaT cell line) using MTT assays according to a standard established procedure. All assays were performed in triplicate.

## 3. Results and Discussion

**Scheme 1:**
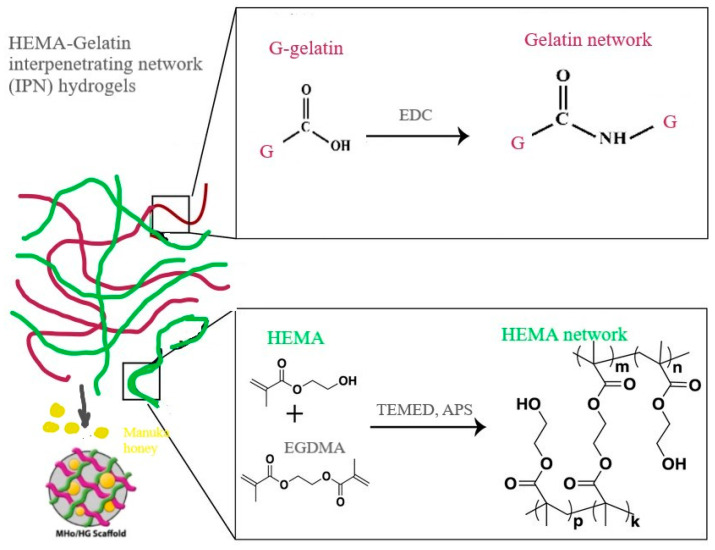
Schematic representations of the procedure to obtain an interpenetrating network hydrogel of 2-hydroxyethyl methacrylate and gelatin (HG IPN) loaded with Manuka honey (MHo/HG scaffolds).

Hybrid hydrogel scaffolds were designed with synthetic 2-hydroxyethyl methacrylate (H) monomer and natural origin polymer gelatin (G) (in the form of interpenetrating hydrogel networks (IPN)) loaded with bioactive agent Manuka honey (MHo) by polymerization/crosslinking reactions (Scheme 1).

### 3.1. Structural Features of MHo/HG Hybrid Hydrogel Scaffolds

**Figure 2:**
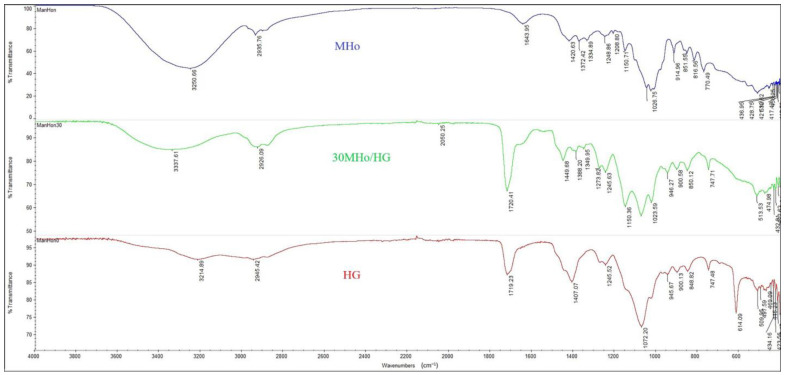
FTIR spectra of MHo, HG hydrogel scaffold, and 30MHo/HG hybrid hydrogel scaffolds.

Fourier transform infrared spectroscopy (FTIR) was used to reveal the main detectable bands for MHo, HG, and Mho/30HG samples (Figure 2) and to prove the incorporation of Manuka honey. Specific peaks originating from HEMA are O–H vibrations around 3200–3340 cm^−1^, C–H around 2920–2945 cm^−1^, and C=O at 1720 cm^−1^, which are attributed asymmetric and symmetric stretching vibrations, methylene stretching, and a carbonyl group [54]. Bands typical of gelatin are C=O stretching around 1700–1600 cm^−1^ for amide I, N–H definition around 1550–1400 cm^−1^ for amide I, and 1240–670 cm^−1^ for amide III [55]. Manuka honey FTIR spectra show five regions. Region 1 around 2800–3000 cm^−1^ corresponds to C–H stretching of carbohydrates and O–H stretching of carboxylic acid, as well as NH_3_ stretching of free amino acids. Region 2 around 1660–1700 cm^−1^ is caused by O–H stretching/bending of water and C=O stretching, mainly from carbohydrates and N–H bending of amide I. Region 3 around 1175–1540 cm^−1^ corresponds to O–H stretching/bending, C=O and C–H stretching of carbohydrates, and C=O stretching of ketones. Region 4 around 940–1175 cm^−1^ corresponds to C–O and C–C stretching of carbohydrates and ring vibration, mainly from carbohydrates. Region 5 around 700–940 cm^−1^ is caused by an anomeric region of carbohydrate C–H bending and ring vibration of carbohydrates [56,57,58,59]. Bands showing the incorporation of Manuka honey are located in the region of 1449–1023 cm^−1^ for the 30MHo/HG sample. 

### 3.2. Porosity of MHo/HG Hybrid Hydrogel Scaffolds

**Figure 3:**
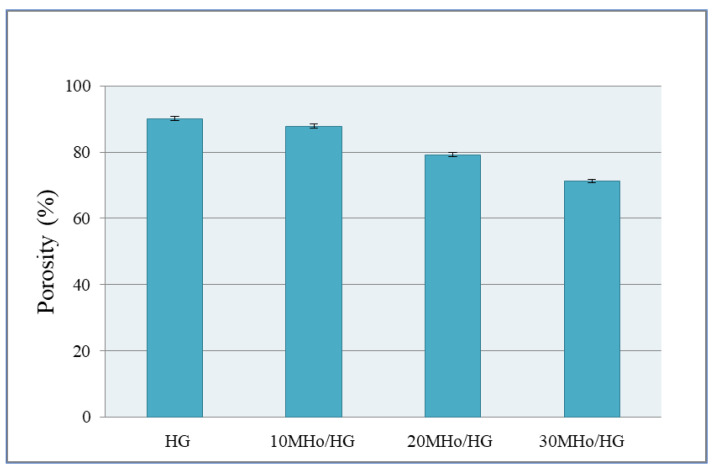
The porosity of MHo/HG hybrid hydrogel scaffolds.

High porosity is a significant scaffold feature for biomedical purposes. The establishment of a porous scaffold structure has a favorable effect on cell attachment, differentiation, proliferation and vascularization processes, oxygen supply, and nutrient flow for a successful tissue regeneration process [60]. Cells need free space to settle, which they get inside pores that are interconnected; in that space, the regeneration process takes place, i.e., tissue growth takes place undisturbed. Our results show that Manuka honey content influences the porosity of prepared hybrid scaffolds. The obtained porosity data are in the range of 71.25–90.09% for the synthesized hybrid hydrogel scaffolds (Figure 3). The sample without honey (HG) has the highest porosity value of 90.09%. The porosity of the hybrid hydrogel scaffolds decreased as the proportion of Manuka honey increased. This phenomenon can be explained by honey filling the free space in the scaffold pores, resulting in decreased porosity. Analysis of the obtained values emphasizes that the synthesized hybrid scaffolds are highly porous, which is adequate for biomedical applications. Because Manuka honey is water-soluble and during dissolution (at the site of application as a scaffold for tissue regeneration, where there is always the presence of body fluids), it frees up space inside the pores due to dissolution, allowing access for cells to populate and spread. According to this hypothesis, Manuka honey dissolves upon interaction with fluid (body fluids), freeing up space within the polymeric network for cells to colonize and spread within the scaffold. Therefore, the sample with 30% Manuka honey is the most favorable for tissue regeneration application. Manuka honey is a very convenient agent for healing, especially for dermal treatments.

### 3.3. Swelling Features of MHo/HG Hybrid Hydrogel Scaffolds

**Figure 4:**
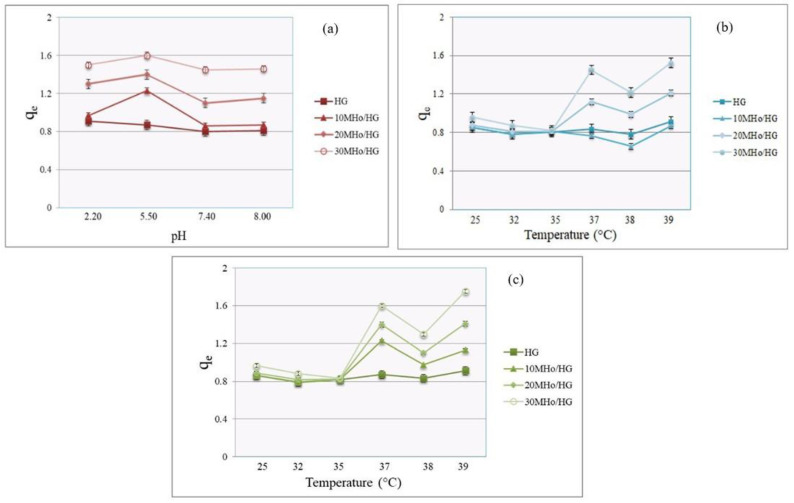
(**a**) pH-dependent swelling performance of MHo/HG; (**b**) temperature-dependent swelling performance of MHo/HG in a pH of 7.40 buffer; and (**c**) temperature-dependent swelling performance of MHo/HG in a pH of 5.50 buffer.

Swelling performance is a very important property of hydrogel scaffolds, providing information about fluid absorption capacity. For biomedical applications, the equilibrium fluid amount of a hydrogel is connected with its ability to absorb body fluid and transfer nutrients to cells and metabolism components through the scaffold [61]. The equilibrium degree of swelling is a key parameter that represents the swelling process. Swelling tests were carried out in buffers with a pH range of 2.20–8.00 at 37 °C and in a temperature range of 25−39 °C in buffers with a pH in the range of 7.40–5.50. Figure 4a–c represents the data for pH and temperature swelling profiles of the designed HG hydrogel scaffold and MHo/HG hybrid hydrogel scaffold samples. Samples show pH- and temperature-dependent swelling behavior. Considering pH influence, the swelling results indicate that the net 2-hydroxyethyl methacrylate/gelatin hydrogel scaffold recorded the lowest equilibrium degree of swelling (q_e_), with a linearly decreasing trend for q_e_−pH dependence in the range of 2.20−8.00 at 37 °C, i.e., pH-dependent behavior was not detected for HG hydrogel scaffolds. When Manuka honey was loaded into the HG scaffold, it imparted specific q_e_-pH dependence of hybrid hydrogel scaffolds. q_e_ values for MHo/HG samples are higher than those of the net HG sample. The same q_e_−pH dependence profile was observed for all MHo/HG samples. q_e_ increases from a pH of 2.20, reaches a maximum value at 5.50, then decreases at a pH of 7.40, with another slight decrease at a pH of 8.00 (q_e_ values are in the range of 0.80−1.60). As the honey content increases, so does the q_e_. Sample 30MHo/HG shows the highest q_e_ value at a pH of 5.50. These types of hybrid hydrogel scaffolds contain hydrophilic groups (–OH, –COOH, and –NH_2_), which enhance the permeability and hydrophilic capacity of the hydrogel network. An increase in hydrophilicity increases the tendency of water molecules to infiltrate the hydrogel network, which results in higher swelling capacity. Honey consists mostly of highly hydrophilic moieties, so its loading on hydrogel scaffolds increases the hydrophilic character of the scaffold, leading to more intense water absorption. The q_e_ values of MHo/HG samples are higher than those of the HG sample and increase with increasing MHo content [62]. The loading of bioactive Manuka honey into the 2-hydroxyethyl methacrylate/gelatin hydrogel scaffold achieved specific pH-dependent swelling behavior of the MHo/HG.

The influence of the temperature interval from 25 °C to 39 °C on the swelling properties of HG and MHo/HG is shown in Figure 4b,c. All hydrogel scaffolds show specific temperature-dependent swelling behavior (q_e_ values are in the range of 0.654−1.753). At the beginning of the temperature interval from 25 °C to 35 °C, an almost linear (slightly decreasing) dependence of q_e_ on temperature can be observed. With an increase in temperature to 35 °C and up to 37 °C, a significant increase in q_e_ occurs. As the honey content in the samples increases, there is also a significant increase in q_e_ in this temperature interval. Furthermore, with increasing temperature from 37 °C to 38 °C, q_e_ values decrease. For the last temperature interval from 38 °C to 39 °C, the highest increase in q_e_ was noticed. It is observed that q_e_ values in a buffer with a pH of 5.50 are higher compared to those in a buffer with a pH of 7.40, depending on the honey fraction. The swelling profiles are the same for all MHo/HG samples in terms of temperature dependence. The hydrophilic–hydrophobic balance is a phenomenon that can explain the swelling behavior of the scaffolds in this temperature interval. At lower temperatures, hydrophobic interactions are more dominant, so the scaffolds show lower swelling. With an increase in temperature, that balance changes, and hydrophilic interactions are more dominant, so swelling increases. In the interval of 37–39 °C, the presence of the gelatin network comes to the fore, which results in decreased swelling, followed by an increase in swelling, which is caused by the segmental mobility of parts of the network originating from the presence of gelatin. This behavior leads to the formation of free space between the network polymeric chains, and water molecules can enter the hybrid scaffolds, causing swelling capacity changes, i.e., specific temperature-dependent swelling occurs [63]. According to our earlier studies, the HEMA component in hydrogels does not contribute to temperature-dependent swelling [64]. The samples containing honey compared with the HG sample show different values of q_e_ as a function of pH and T. It should be emphasized that the q_e_ value is 1.2 for 10MHo/HG at a pH of 5.50, but q_e_ is 0.8 for the same sample at a pH of 7.40; a difference of 0.4 is enough to cause different behavior of 10MHo/HG at different pH values. A sufficient difference in swelling was also shown in the dependence of q_e_ on T. Changes in pH and temperature are the most important stimuli for various biomedical applications (including wound healing). The correlations of swelling with pH and temperature are important properties of hydrogel scaffolds. The sample with the highest content of Manuka honey (30MHo/HG) shows the highest q_e_ value in a solution with a pH of 5.50 and at a temperature of 39 °C, which are pathophysiological conditions indicating infections and diseases. Therefore, this sample is expected to show the best therapeutic properties for the treatment of diseased tissues and is recommended for biomedical applications [4,5,6,7].

### 3.4. In Vitro Degradation Behavior of MHo/HG Hybrid Hydrogel Scaffolds

**Figure 5:**
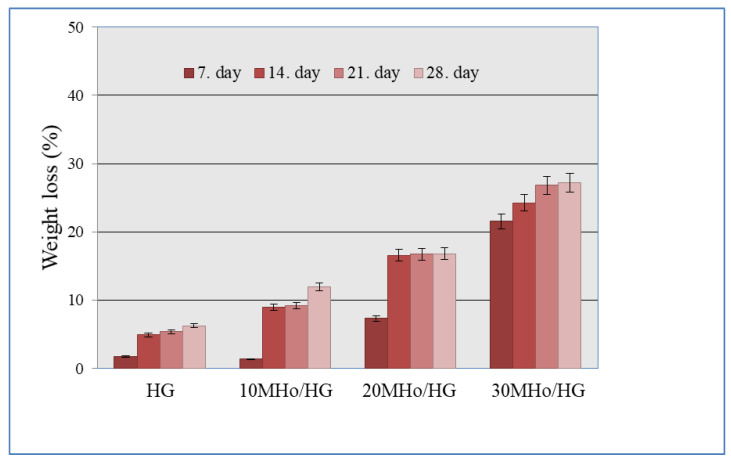
In vitro degradation of MHo/HG hybrid hydrogel scaffolds over 4 weeks.

Degradability is an important feature to validate the suitability of biomaterial for biomedical applications. The scaffold degradation process provides new space for tissue regrowth and induces the regeneration process. Tunable degradation is possible with synthetic hydrogels depending on chemical composition and the ratio of components. Hydrogels of natural origin are intrinsically degradable and also impact degradability. These combinations are the beneficial synergy that makes it possible to adjust the degradable properties of hybrid hydrogel scaffolds. Because a scaffold provides mechanical support for cell growth, the degradation process should be time-dependent with a controlled rate that guarantees mechanical support during the regeneration process [65]. In vitro degradability of HG and MHo/HG was tested in a phosphate buffer for 4 weeks. The obtained data are presented in Figure 5. It is obvious that composition influences degradation behavior. The net HG scaffold shows the lowest weight loss value. Loading MHo into the HG scaffold accelerates the degradation process. As the honey fraction increases (10−30%), so does MHo/HG weight loss (6.27% to 27.18%). The 30MHo/HG sample shows the highest degradation. This behavior indicates that honey is a component of the hybrid scaffolds that affects the degradation process due to its highly hydrophilic character, making it easier for water molecules to penetrate the hydrogel network and accelerate degradation.

### 3.5. Biocompatibility Assays of MHo/HG Hybrid Hydrogel Scaffolds

**Figure 6:**
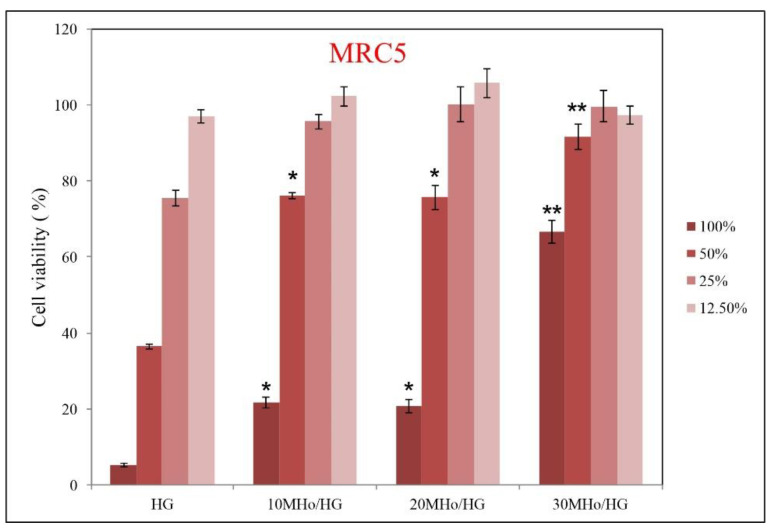
Cell viability for human fibroblasts upon treatment with extracts of MHo/HG (percentage of living cells compared to the DMSO-treated control using a *t*-test, * *p* ≤ 0.05, ** *p* ≤ 0.01).

**Figure 7:**
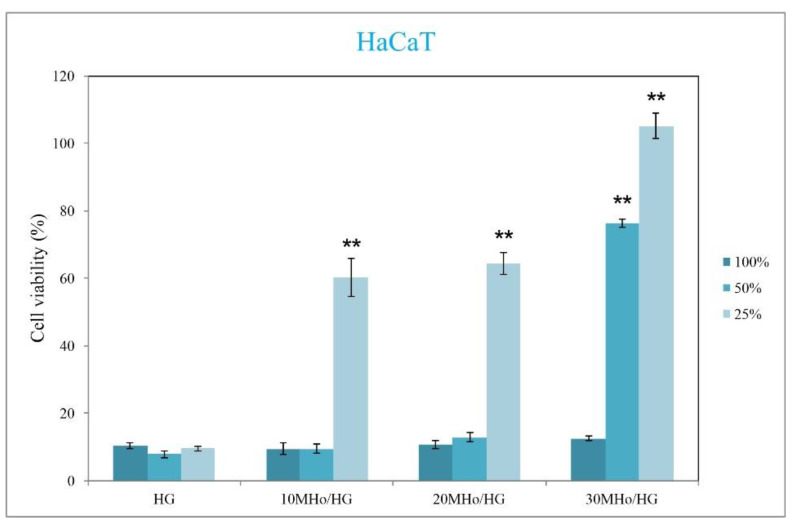
Cell viability for human keratinocytes upon treatment with extracts of MHo/HG (percentage of living cells compared to the DMSO-treated control using a *t*-test, ** *p* ≤ 0.01).

Biocompatibility assessment is an essential procedure for preclinical probes of biomaterials intended for biomedical purposes [47,66,67,68,69,70,71,72]. In vitro biocompatibility tests provide data on whether the tested biomaterial or any components that did not react (leachables) in cell interaction assays cause cell death or interfere with cell functions. The biocompatibility of MHo/HG was evaluated for interaction with normal human fibroblasts (MRC5) and normal human keratinocytes (HaCaT). Data from these assays are shown in Figure 6 and Figure 7. The obtained data indicate that Manuka honey content influences cell viability for both cell lines. Better cell viability was achieved for scaffolds interacting with the fibroblast cell line, whereas keratinocytes showed lower cell viability. The sample with the highest honey content (30%) shows the best biocompatible properties for both tested cell lines. Therefore, this sample is the most suitable for applications as a scaffolding biomaterial for tissue regeneration. Interestingly, material extracts containing no Manuka honey inhibited keratinocyte proliferation quite efficiently (Figure 7), and dose dependence was observed in the case of MRC5 cells (Figure 6). Human HaCaT keratinocyte cells are immortal epithelial cells that are susceptible to malignant transformation [73] and have been previously shown to be more sensitive than MRC-5 cells in the case of compounds including hexamidine, synthalin, para-guanidino ethylphenol [74]. Nevertheless, the MHo component showed a beneficial effect on the proliferation of both cell lines. In comparison, the selection of honey samples tested directly on MRC5 cells showed IC_50_ values of 10–50 mg/mL [75]. Previous studies have shown that the MHo component improves wound healing through the stimulation of cytokine induction [76,77].

## 4. Conclusions

Our research revealed interesting and advantageous features of newly designed bioactive Manuka honey/2-hydroxyethyl methacrylate/gelatin hybrid hydrogel scaffolds. Manuka honey content determines all tested scaffolding properties. Structural characteristics confirmed the loading of Manuka honey into 2-hydroxyethyl methacrylate/gelatin hydrogel scaffolds, with porosity values in the range of 71.25% to 90.09 %. It is known that Manuka honey has appropriate properties for treatments, especially for dermal purposes. Manuka honey is water-soluble, and during dissolution (at the site of application as a scaffold for tissue regeneration, where there is always the presence of body fluids), it frees up space inside the pores due to dissolution, allowing access for cells to populate and spread. Hybrid hydrogel scaffolds showed pH- and temperature-dependent swelling performances and favorable absorption capacity, with q_e_ values proportional to the MHo amount, showing the highest q_e_ values for sample 30MHo/HG. In vitro degradation studies exhibited scaffold weight loss in the range of 6.27% to 27.18% during four weeks. In vitro biocompatibility probes on healthy human fibroblast (MRC5 cells) and keratinocyte (HaCaT cells) cell lines by MTT test indicated that cell viability depends on the Manuka honey content and that Manuka honey improves cell viability. The results indicate that the use of 2-hydroxyethyl methacrylate/gelatin hybrid hydrogel scaffolds loaded with Manuka honey has the potential for medical use. The advantageous properties of MHo/HG hybrid hydrogel scaffolds for biomedical applications can be realized by modulating Manuka honey content. The simplified design strategy and easy creation of MHo/HG hybrid scaffolds have significant implications for possible clinical probes. This research shows the medical potential for the obtained hybrid scaffolds. Therefore, additional research on these novel scaffolds in the form of in vivo assays and clinical studies is required. The goal of all research considering the design of hydrogel scaffolds is to synthesize ideal scaffolds that meet all requirements when it comes to specific medical applications (regeneration and healing of different tissue types). The use of Manuka honey in treatment is an inexhaustible research topic, as shown by the latest published papers.

## Figures and Tables

**Table 1:** Components and marks of hydrogel scaffold samples.

Sample	Component 1	Component 2	Component 3	Crosslinker for HEMA	Crosslinker for Gelatin	Initiator/Activator
HG	HEMA	Gelatin	–	EGDMA	EDC	APS/TEMED
10MHo/HG	HEMA	Gelatin	MHo	EGDMA	EDC	APS/TEMED
20MHo/HG	HEMA	Gelatin	MHo	EGDMA	EDC	APS/TEMED
30MHo/HG	HEMA	Gelatin	MHo	EGDMA	EDC	APS/TEMED

## Data Availability

Not applicable.
